# Exploring functional core bacteria in fermentation of a traditional Chinese food, *Aspergillus*-type douchi

**DOI:** 10.1371/journal.pone.0226965

**Published:** 2019-12-30

**Authors:** Huilin Yang, Lin Yang, Ju Zhang, Hao Li, Zongcai Tu, Xiaolan Wang

**Affiliations:** Key Lab of Protection and Utilization of Subtropic Plant Resources of Jiangxi Province, Jiangxi Normal University, Nanchang, China; Tallinn University of Technology, ESTONIA

## Abstract

Douchi is a type of traditional Chinese flavoring food that has been used for thousands of years and is produced by multispecies solid-state fermentation. However, the correlation between the flavor, the microbiota, and the functional core microbiota in *Aspergillus*-type douchi fermentation remains unclear. In this study, Illumina MiSeq sequencing and chromatography were used to investigate the bacterial community and flavor components in *Aspergillus*-type douchi fermentation. The dominant phyla were Firmicutes, Proteobacteria, and Actinobacteria, and the dominant genera were *Weissella*, *Bacillus*, *Anaerosalibacter*, *Lactobacillus*, *Staphylococcus*, and *Enterococcus*. A total of 58 flavor components were detected during fermentation, including two alcohols, 14 esters, five pyrazines, three alkanes, four aldehydes, three phenols, six acids, and five other compounds. Bidirectional orthogonal partial least square modeling showed that *Corynebacterium_1*, *Lactococcus*, *Atopostipes*, *Peptostreptococcus*, norank_o__AKYG1722, *Truepera*, *Gulosibacter*, norank_f__Actinomycetaceae, and unclassified_f__Rhodobacteraceae are the functional core microbiota responsible for the formation of the flavor components during douchi fermentation. This is the first study to investigate the functional core microbiota in douchi fermentation using Illumina MiSeq sequencing and chromatographic techniques. Our findings extend our understanding of the relationships between flavor, the microbiota, and the functional core microbiota during *Aspergillus*-type douchi fermentation.

## Introduction

Douchi is a kind of traditional Chinese flavoring produced with multispecies solid-state fermentation, which involves multiple microorganismal species spontaneously grown in medium such as beans, tofu, or straw. The microbiota in the medium drives the fermentation process and produces the flavor components after a period of time[1].

In general, douchi is produced in two stages, koji making and fermentation [2]. During koji making, the microorganisms (especially fungi) that produce the various functional enzymes that play important roles in the degradation of key ingredients, such as lipids, proteins, carbohydrates, and other functional constituents, for subsequent fermentation[3]. Fermentation is in initiated with a succession of microbiota, which is reproducible and well balanced, even after a long period and repetitive fermentation processes. This stage of douchi production creates its characteristic flavor compounds and nutritional content, which are determined by the succession of microbiota.

With technological developments, the microbiota and flavor components in fermented foods, such as kimichi[4], Chinese rice wine[5], and grape wine[6], have been extensively investigated, and the relationships between the microbiotal succession and the metabolic changes that occur during fermentation have been determined. However, only a few studies[1] have investigated the functional core microbiota in fermented foods based on a correlation analysis of the microbiota and the flavor compounds they produce, which may be attributable to the complexity of the microbiota and the variability in the flavor components. The bidirectional orthogonal partial least square (O2PLS) method[7, 8] is a systems biology approach based on data collected from different analytical platforms, and uses advanced statistics to determine potential associations[9]. In a previous study, Lambert et al.[10] used this method to assess the effects of supplementation with yellow pea fiber on weight loss and the gut microbiota in an overweight or obese adult population. Li et al.[11] used an O2PLS approach to integrate metabonomic and microbiology analyses of the metabolic interactions between microbiota and host rats, and Wang et al. used it to investigate the functional core microbiota in the association between microbial succession and flavor in Chinese vinegar fermentation[1].

In the present study, the bacterial community involved in a fermentation process was characterized with Illumina MiSeq sequencing, the flavor components were determined with chromatography, and both were analyzed with multivariable statistics. The correlation between the microbiota and the flavor components was investigated with the O2PLS approach, and the functional core bacteria were identified. Our results identify the functional core microbiota based on the dominant microbes present and the flavor compounds produced during the douchi fermentation process.

## Materials and methods

### Sample collection and preparation

A total of eight samples, collected during the douchi fermentation process in the workshop of Daoxiangyuan Corporation (Nanchang, Jiangxi Province, China), were examined in this study. All the samples were obtained from the same site. Triplicate samples (100 g each) were collected in fermentation day 1 (F1), fermentation day 2 (F2), fermentation day 3 (F3), fermentation day 4 (F4), fermentation day 5 (F5), fermentation day 6 (F6), fermentation day 7 (F7), and fermentation day 8 (F8) and mixed together to reduce errors. The samples were immediately stored at −80 °C before subsequent analysis.

### DNA extraction

DNA was extracted during the koji making and fermentation stages with the Omega E.Z.N.A^®^ Soil DNA Kit (Feiyang Biotech Co., Ltd, Guangzhou, China), according to the manufacturer’s instructions, without modification[12]. The DNA quality was checked with 0.8% agarose gel electrophoresis, and the DNA was stored at −80°C before further analysis.

### Illumina MiSeq sequencing

The V4 region of 16S rRNA was amplified with primers 515F (5′-GTGCCAGCMGCCGCGGTAA-3′) and 806R (5′-GGACTACHVGGGTWTCTAAT-3′). The 5′-barcoded amplicons were generated by PCR with Ex Taq HS (TaKaRa Bio Inc., Shiga, Japan) under the following conditions: 95 °C for 2 min, followed by 35 cycles of 95 °C for 30 s, annealing at 55 °C for 1 min, and extension at 72 °C for 1 min; with a final extension at 72 °C for 10 min. The amplicons were pooled in equimolar amounts and sequenced with the Illumina MiSeq platform and MiSeq Reagent Kit v1 (Illumina, Inc., Santiago, CA, USA) at the Beijing Genomics Institute (Shenzhen, China).

Clean data were obtained with scripts written in-house, as follows: (1) sequences containing > 1 ambiguous bases (N) were removed; (2) the completeness of barcodes and adaptors was confirmed; and (3) sequences of < 100 bp were removed. All the 250-bp pair-end sequence reads were connected using the COPE software (Connecting Overlapped Pair-end, V 1.2.1)[13] to merge the read pairs from DNA fragments into tags. Further data processing was performed as previously described[14–19] and included the removal of sequencing noise using the pre.cluster tool in the MOTHUR software package v. 1.31.2[20] and *de novo* chimera detection and removal in UCHIME v. 4.2. Operational taxonomic units (OTUs) were determined with MOTHUR, with a 97% sequence identity threshold[20]. The 16S rRNA reads were assigned with 16S rRNA training set 9 in the RDP database using a local BLAST search.

### Determination of volatile components generated during douchi fermentation

The douchi samples were freeze-dried and powdered, and 3 g of the douchi powder was added to a 20 mL header flask containing 6 mL of distilled water, which was then sealed with a polytetrafluoroethylene septum. A 50/30 μm divinylbenzene/carboxen/polydimethylsiloxane extraction head was used, and the samples were uniformly heated on a magnetic stirrer at 60 °C for 30 min. An activated solid phase microextraction (SPME) head was inserted through the septum (activated at 270 °C for 1 h), the fiber head was ejected, and the headspace gas was adsorbed for 30 min. After the extraction temperature reached 60 °C, the samples were inserted into the gas chromatograph inlet for 5 min. The gas chromatography oven temperature was maintained at 40 °C for 5 min, increased to 90 °C at the rate of 5 °C/min, and then increased to 230 °C at the rate of 12 °C/min, where it was held for 8 min. The ion source and interface temperature were set to 250 °C and 200 °C, respectively. The mass detector was operated in the positive ion electron impact ionization (EI+) mode at 70 eV in the range of 33–450 m/z.

### Determination of free amino acids produced during douchi fermentation

In the pretreatment process, 2 g of each douchi sample was added to 2 mL of 8% sulfosalicylic acid solution, homogenized, and centrifuged for 20 min at 16,000 r/min. The supernatant was filtered with a 0.22 μm water filter and stored at −70 °C. The contents of free amino acids (AA) were determined with an amino acid analyzer (Hitachi L-8900)[21] using a separation column (4.6 mm × 60 mm, resin 2619 #) at a column temperature of 53 °C, a reaction temperature of 98 °C, detection wavelengths of 570 and 440 nm, a flow rate of 0.225 mL/min, and a pump pressure of 110 kg/cm^2^.

### Statistical analysis

The sequences were processed and analyzed on the QIIME platform (version 18)[22]. The alpha diversity indices, such as Simpson’s diversity index, Chao 1, and Shannon’s diversity index, were analyzed with the MOTHUR package[23]. A principal coordinates analysis (PCoA) and clustering analysis based on the microbiota were performed with the R 3.5.0 software[24]. A clustering analysis and hierarchical cluster analysis (HCA) based on the flavor compounds and the correlation analysis of the microbiota and flavor compounds were performed with SIMCA-P version 14.0 (Umetrics, Umea, Sweden) to examine the intrinsic variations in the data and to identify outliers[25]. Statistically significant differences were detected with the nonparametric Mann–Whitney test, Adonis analysis, and multivariate analysis of variance, which were performed in MATLAB R2014a (MathWorks, Natick, MA, USA)[26–28]. p values of < 0.05 were considered to indicate statistical significance.

### Nucleotide sequence accession numbers

Sequences reported in this paper are available in the SRA database under accession number SRP154181 (https://www.ncbi.nlm.nih.gov/sra/SRP154181).

## Results and discussion

### Bacterial community structure analysis

A total of 1,357,004 high-quality sequences were used for the taxonomic classification, which generated a total of 423 bacterial OTUs with 97% similarity and > 0.995 coverage. The rarefaction curves for the observed species indices and Simpson’s diversity indices for bacteria are presented in S1 Fig. As can be seen, although the observed species index curves are not parallel to the x-axis, Simpson’s indices are saturated and parallel to the x-axis, suggesting that the sequences represent the majority of the microbiota involved in the douchi fermentation process. Furthermore, the species index (Table 1) shows an increasing trend from F1 to F8, indicating that the abundance and diversity of the microbial community increased during the douchi fermentation process.

**Table 1:** Sample information, sequence abundance, and microbial diversity.

Samples	Sobs	Shannon	Simpson	Ace	Chao 1	Coverage
F1	193	2.61	0.12	243.45	234.40	0.9994
F2	172	2.13	0.22	226.00	223.13	0.9994
F3	319	3.20	0.09	342.28	332.06	0.9996
F5	252	2.94	0.13	288.11	286.17	0.9996
F6	281	3.39	0.06	322.65	330.00	0.9994
F7	307	3.29	0.09	345.93	345.90	0.9994
F8	345	3.07	0.17	366.34	364.03	0.9996
Mean±SD	272.38±62.02	3.00±0.43	0.12±0.05	306.81±50.21	304.26±51.73	0.9995±0.0001

F1, fermentation day 1; F2, fermentation day 2; F3, fermentation day 3, etc.

**Fig 1:**
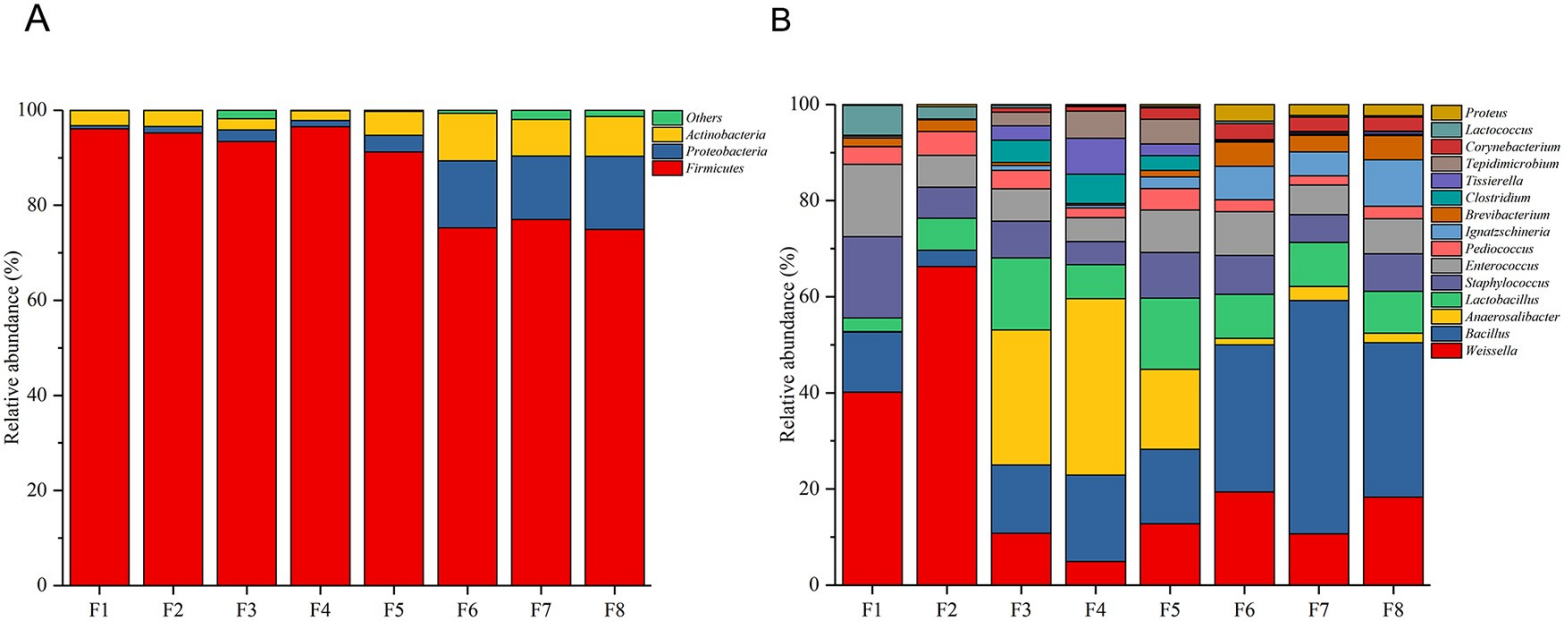
Relative abundances of bacteria at the phylum (a) and genus (b) levels during douchi fermentation.

A total of 15 bacterial phyla were identified during douchi fermentation, among which the dominant phyla (Fig 1a) were Firmicutes (87.12%), Proteobacteria (6.78%), and Actinobacteria (5.29%). Other phyla (< 1%) constituted 0.81% of the total phyla detected. At the genus level, 240 genera were identified during douchi fermentation, among which the dominant genera (Fig 1b) were *Weissella* (19.17%), *Bacillus* (19.44%), *Anaerosalibacter* (10.26%), *Lactobacillus* (8.10%), *Staphylococcus* (7.17%), and *Enterococcus* (6.29%). Other major genera (> 1%) included *Pediococcus* (2.85%), *Ignatzschineria* (2.82%), *Brevibacterium* (2.18%), *Clostridium* (1.69%), *Tissierella* (1.68%), *Tepidimicrobium* (1.67%), *Corynebacterium* (1.52%), *Lactococcus* (1.11%), and *Proteus* (1.03%), The remaining genera detected comprised 11.87% of the total bacterial genera identified. These results are similar to those reported in previous studies. For instance, when Chen et al. examined the key microorganisms involved in the douchi fermentation process, the dominant organisms were Staphylococcaceae, Bacillaceae, Enterococcaceae, Pseudomonadaceae, and Enterobacteriaceae[3]. Similarly, Yang et al. reported that *Staphylococcus* and *Weissella* were the dominant genera, and that *Acinetobacter*, *Bacillus*, *Corynebacterium*, *Lactobacillus*, and *Pseudomonas* were relatively abundant genera (> 1%) during the douchi fermentation process[2].

**Fig 2:**
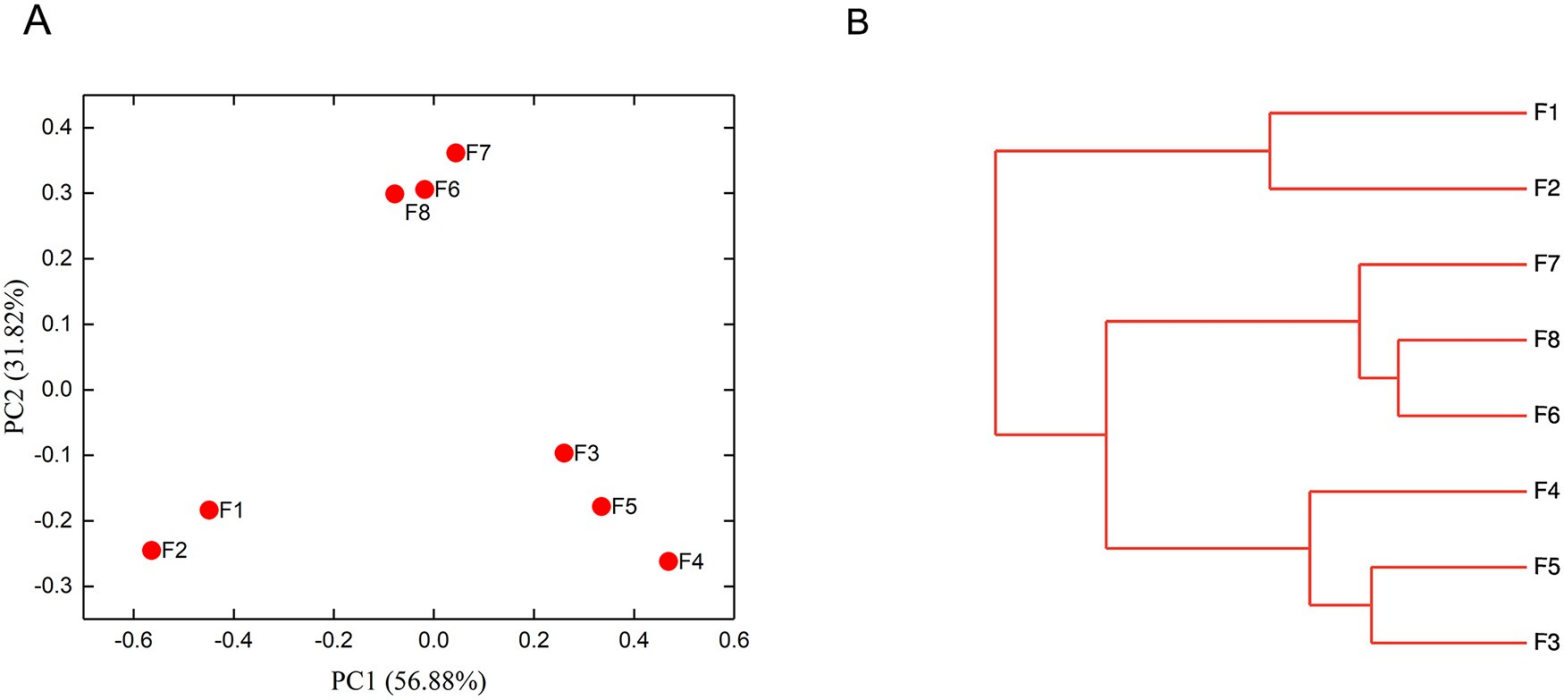
Bacterial structural analysis during the douchi fermentation process. (a) PCoA of the microbial communities, PC1 variance = 56.88%, PC2 variance = 31.82%. (b) Cluster analysis of the bacterial microbiota involved in the douchi fermentation process. Green indicates samples belonging to group 1 and red indicates samples belonging to group 2.

To investigate the similarity in the trends among the samples, we used a principal coordinates analysis (PCoA) and a cluster analysis. The PCoA (Fig 2a) and cluster plots (Fig 2b) showed that the samples grouped into three stages: I, F1–F2; II, F3–F5; and III, F6–F8. However, an Adonis analysis indicated that the grouping results were significant when stages II and III were merged into one section. Therefore, the new grouping based on the Adonis analysis was: group 1, F1–F2, and group 2, F3–F8. This result indicates that the degree of variation (Fs) among the groups was larger than that within each group. The p value between groups 1 and 2 was 0.003, demonstrating that the structures of the groups (group 1 vs group 2) were significantly different. The significant difference (p < 0.05) between group 1 and group 2 mainly resulted from the change in the microbiota during the douchi fermentation process. As shown in Fig 1b, the relative abundances of the genera *Weissella*, *Staphylococcus*, *Enterococcus*, and *Lactococcus* in F1–F2 were 37.44%–63.79%, 6.21%–15.73%, 6.35%–14.01%, and 2.47%–5.82%, respectively. However, in F3–F8, the relative abundances of these genera decreased sharply to 4.41%–16.45%, 4.35%–6.90%, 4.43%–7.90%, and 0.21%–0.39%, respectively, and they even became minor genera. The relative abundances of other genera in F1–F2 were *Bacillus* 3.22%–11.43%, *Anaerosalibacter* 0.01%–0.02%, *Lactobacillus* 2.67%–2.42%, *Ignatzschineria* 0.01%, and *Brevibacterium* 1.67%–2.35%. Interestingly, in F3–F8, the proportions of these genera increased sharply, and some even became dominant genera, with relative abundances of 12.61%–40.01%, 1.18%–32.89%, 6.34%–13.31%, 0.51%–8.00%, and 0.31%–4.27%, respectively.

The dynamic changes in the structure of the microbiota during the douchi fermentation process and the significant differences between the two groups (p < 0.05) might have been caused by the changed environmental conditions during the douchi fermentation process, which included extremely low oxygen, higher temperature, higher salt concentration, and lower humidity[29]. Wang et al.[2] and Chen et al.[30] reported that that high salt concentration, high temperature, low humidity, and submerged fermentation prohibited the growth of microorganisms that could not tolerate the harsh conditions required for douchi fermentation. In our study, genera such as *Bacillus*, *Anaerosalibacter*, and *Lactobacillus* that were anaerobic or thermotolerant bacteria[31–35] increased quickly, although some genera showed decreasing trends in the late stage of fermentation, which might be attributable to interspecific competition. Yang et al. reported that interspecific competition affects the relative microbial abundances during douchi fermentation[2].

### Evaluation of flavor components and multivariable analysis

A total of 58 flavor components were identified during the douchi fermentation process, including 42 volatile flavor compounds (VFs) and 16 AAs. The VFs could be grouped into eight categories: two alcohols (compound nos 1–2), 14 esters (compound nos 3–16), five pyrazines (compound nos 17–21), three alkanes (compound nos 22–24), four aldehydes (compound nos 25–28), three phenols (compound nos 29–31), six acids (compound nos 32–37), and five other compounds (compound nos 38–42). The details of the flavor components are given in Table 2. Although numerous studies have investigated the components that affect the aromas of fermented foods[36–40], research into the flavor components generated during douchi fermentation has been limited. In a previous study, Chen et al. investigated the flavor compounds in liuyang douchi with gas chromatography–mass spectrometry and gas chromatography–olfactometry. They found that the flavor components in douchi included 2-methyl-butanal, ethyl 2-methylbutyrate, isoamyl acetate, 2,6-dimethylpyrazine, 1-octen-3-ol, 2-pentyl furan, benzeneacetaldehyde, phenylethyl alcohol, phenethyl acetate, and phenethyl butyrate, similar to those noted in the present study[41]. Similarly, the AAs detected during douchi fermentation in the present study are consistent with those reported by Chen et al., who investigated the free AA contents in a variety of commercial douchi[3].

**Table 2:** Flavor components generated during douchi fermentation.

ID	Flavor	ID	Flavor
No.1	Ethanol	No.22	Hexamethylcyclotrisiloxane
No.2	Phenethyl alcohol	No.23	Octamethylcyclotetrasiloxane
No.3	Ethyl acetate	No.24	Undecane
No.4	Ethyl isobutyrate	No.25	Isovaleraldehyde
No.5	Ethyl butyrate	No.26	Benzaldehyde
No.6	Ethyl 2-methylbutyrate	No.27	Phenylacetaldehyde
No.7	Ethyl caproate/4-methyl-Pentanoic acid, ethyl ester	No.28	l-Caryophyllene
No.8	Methyl benzoate	No.29	Guaiacol
No.9	2,6-Di-tert-butyl-4-methylphenol	No.30	2-Ethylphenol
No.10	Methyl hexadecanoate	No.31	Cocal
No.11	Ethyl palmitate	No.32	Butyric acid
No.12	Octadecenoic acid methyl ester	No.33	Isovaleric acid
No.13	Methyl linoleate	No.34	DL-2-Methylbutyric acid
No.14	Ethyl oleate	No.35	4-Methylvaleric acid
No.15	9,12-Octadecadienoic acid (Z, Z)-, ethyl ester	No.36	Palmitic acid
No.16	(Z, Z, Z)-9,12,15-Octadecatrienoic acid, ethyl ester	No.37	Linoleic acid
No.17	2,5-Dimethyl pyrazine	No.38	Tetrahydrothiophene
No.18	2,3,5-Trimethylpyrazine	No.39	2-Chloro-4-(4-methoxyphenyl)-6-(4-nitrophenyl) pyrimidine
No.19	3-ethyl-2,5-dimethyl-Pyrazine	No.40	1,3-diphenyl-1-(trimethylsilyioxy)-1-Heptene
No.20	Tetramethylpyrazine	No.41	α-ethylidene-Benzeneacetaldehyde
No.21	3,5-diethyl-2-methyl-Pyrazine	No.42	Ethyl 3-phenylpropionate

**Fig 3:**
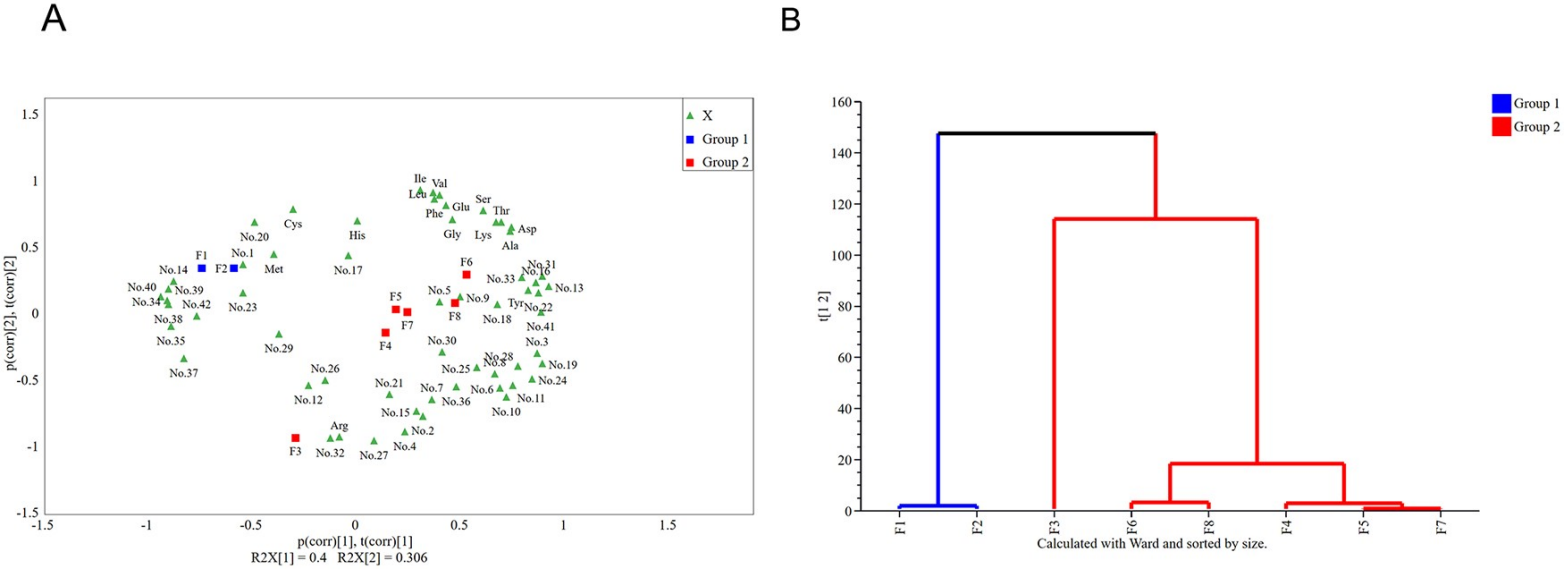
Flavor compound structural analysis during the douchi fermentation process. (a) PCoA of flavor components generated during douchi fermentation. Green box indicates the flavor components, blue triangle indicates group 1, and red triangle denotes group 2. The grouping mode is based on Hellinger distances with 97% similarity. (b) HCA of the flavor components generated during douchi fermentation.

PCoA was used to examine the distribution of the flavor components during the douchi fermentation process. The results (Fig 3a) showed that the first two components R^2^X(cum) explained 70.6% of the variables, and that the cross value, validated Q^2^, for each component was more than the threshold for the component (limit), suggesting significant components for analysis (S1 Table). Moreover, the projected coordinates of the metabolites in PC1 were consistent with the trends in flavor component production during the douchi fermentation process. HCA (Fig 3b) demonstrated that douchi fermentation could be divided into two groups: group 1, F1–F2, and group 2, F3–F8. Interestingly, the grouping mode based on flavor components was consistent with that based on the microbiota during douchi fermentation. Few flavor components were detected in group 1 (F1–F2) fermentation, and included two AAs and 12 VFs: one alcohol, one ester, one pyrazine, one alkane, one phenol, three acids, and four other compounds. This indicates that in the early stage of fermentation, the bacteria must grow and adapt to their harsh environment. Marina et al.[42] used combined metabolic and transcriptomic profiling to investigate how *L*. *lactis* subsp. *cremoris* MG1363 adapts to oxidative stress. Jinhee et al.[43] investigated the dynamic changes in the microbial community in the Korean traditional fermented food kimchi and found that the microbiota grew at a lower temperature in the early period of fermentation, before the period of higher-temperature fermentation as the microbes adapted to the fermentation environment. This result is consistent with our hypothesis that the microbiota must adapt to the new environment, including the increased temperature, reduced humidity, extremely low oxygen, and the addition of salt. Therefore, most of the flavor compounds were not produced in the early stage of douchi fermentation. However, the majority of these components were detected in the following stage of fermentation (F3–F8), including 14 AAs and 30 VFs: one alcohol, 13 esters, four pyrazines, two alkanes, four aldehydes, two phenols, three acids, and one other compound (Fig 3a). This indicates that most of the flavor components were produced by group 2. Thus, the groupings based on the flavor compounds and the microbial community structures suggest that most of the flavor components were produced in the later stage of fermentation, which is consistent with the findings of previous studies[44, 45]. The later fermentation stage played an key role in the formation of the flavor compounds, which is consistent with the results of John et al.[46], who demonstrated that most of the flavor compounds and/or volatiles were detected in the later stage of bread fermentation. Ye et al.[47] investigated the changes in the profiles of volatile compounds and amino acids during cider fermentation from a dessert variety of apple, and found that the maximum concentrations of esters appeared in the later stage of fermentation.

### Correlation analysis of microbiota and flavor components during douchi fermentation

**Fig 4:**
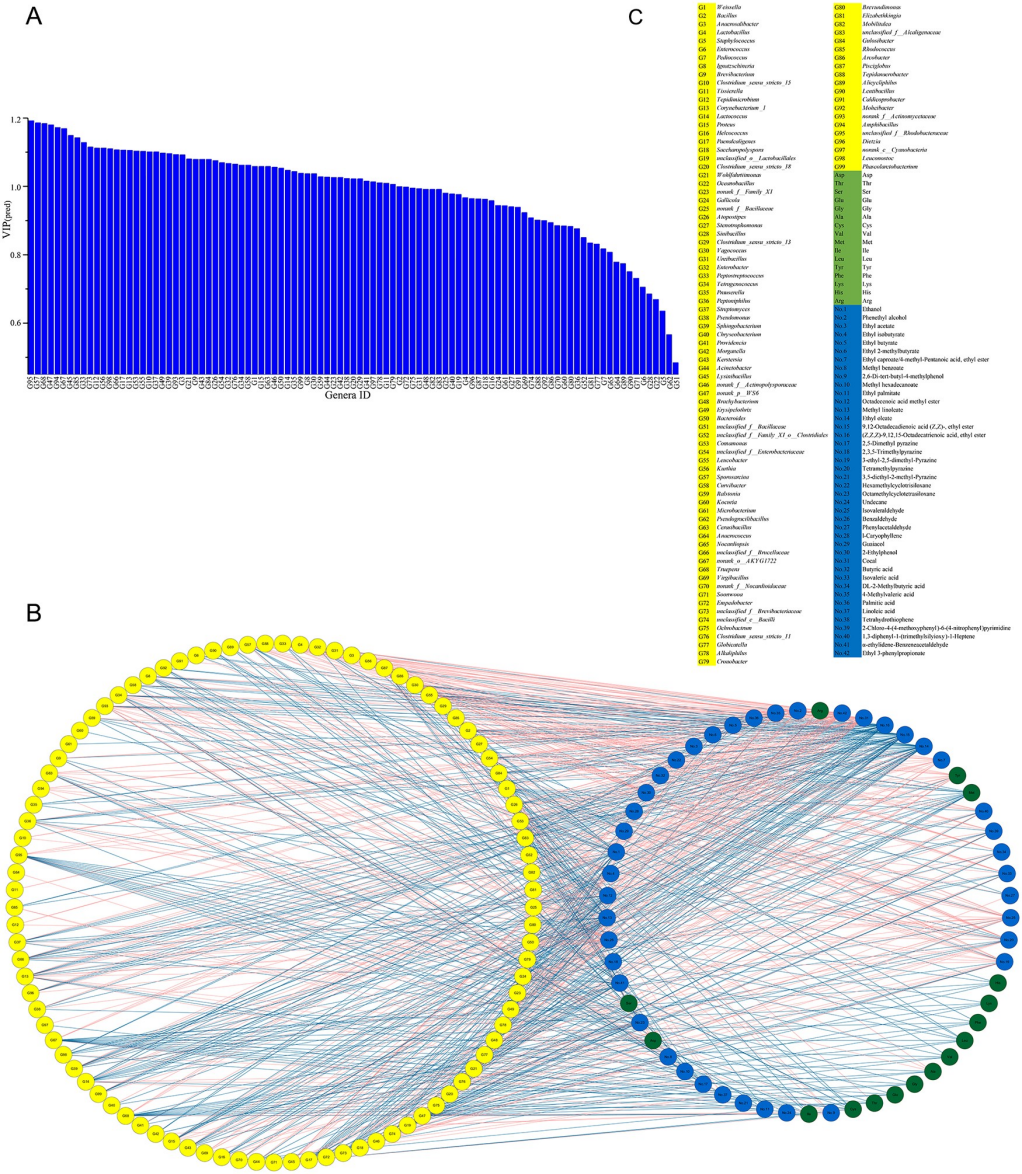
Correlation between microbiota and flavor components in the douchi fermentation process, evaluated with the O2PLS method. (a) VIP(pred) plot of the microbiota correlated strongly with flavor components (VIP(pred) > 1.0). (b) Network of correlations between microbiota and flavor components during douchi fermentation. The left-side circle represents genera (|*p*| > 0.7); the right-side circle denotes flavor components; red line between microbiota and flavor components indicates positive correlation (p > 0.7); and blue line shows negative correlation (p < −0.7). (c) The details of the microbiota and the flavor components.

The O2PLS method was used to evaluate the correlation between the microbiota and flavor components detected during the douchi fermentation process. In this model, the parameters Q^2^ and R^2^ were 0.332 and 0.851 (O2PLS modeling of S1 Table), respectively, indicating that this method was suitable for the analysis of and prediction from these data, which was similar with the previous paper[1]. The VIP_(pred)_ vector (VIP value for predictive components) for the microbiota was 0.49–1.19 (Fig 4a), and 57 genera (VIP_(pred)_ > 1.0) (S1 Table) had a significant effect on the flavor components. Based on the correlation coefficients between the bacterial community and the flavor components, 51 genera correlated strongly (|*p*| > 0.7) (S1 Table) with both AAs and VFs, 53 genera correlated strongly with AAs, and 92 genera correlated strongly with VFs (Fig 4b). Furthermore, the genera norank_p__WS6, norank_o__AKYG1722, *Truepera*, and unclassified_f__Rhodobacteraceae produced most of the AAs, whereas the genera *Atopostipes*, *Peptostreptococcus*, and *Tetragenococcus* produced 15, 15, and 18 VFs, respectively. Most of the major genera correlated with a variety of flavor components (> 5), and only a few of the major genera (eight) correlated with one flavor component. The detailed correlations (|*p*| > 0.7) between the microbiota and flavor components are shown in S1 Table.

The genera norank_p_WS6, norank_o_AKYG1722, *Truepera*, and unclassified_f__Rhodobacteraceae were important contributors of AAs during douchi fermentation. In a previous study, Dajanta et al. classified the taste of AAs into three types: umami-taste AAs (Glu + Asp), sweet-taste AAs (Ala + Gly + Ser + Thr), and bitter-taste AAs (Arg + His + Ile + Leu + Met + Trp + Tyr + Val)[3]. In the present study, Glu, Ala, Leu, Asp, Val, Lys, Ser, Phe, and Gly were the dominant AAs in douchi fermentation, and the genera norank_p__WS6, norank_o__AKYG1722, *Truepera*, and unclassified_f__Rhodobacteraceae (|*p*| > 0.8) correlated strongly with the umami-taste AAs, whereas *Anaerosalibacter*, *Clostridium_sensu_stricto*_15, *Prauserella*, *Lysinibacillus*, norank_p__WS6, unclassified_f__Enterobacteriaceae, *Sporosarcina*, *Ralstonia*, norank_o__AKYG1722, *Truepera*, *Clostridium_sensu_stricto*_11, *Rhodococcus*, unclassified_f__Rhodobacteraceae, and *Phascolarctobacterium* (|*p*| > 0.8) correlated strongly with the bitter-taste AAs. Interestingly, none of the detected genera (|*p*| > 0.8) correlated strongly with the sweet-taste AAs (Fig 4b). Chen et al.[48] reported that filamentous fungi, yeasts, *Bacillus*, *Staphylococcus*, *Enterobacter*, and lactic acid bacteria correlated positively with free amino acids, and Wang et al.[1] demonstrated that *Acetobacter*, *Aspergillus*, *Lactobacillus*, *Enhydrobacter*, *Roseomonas*, *Sphingobacterium*, *Staphylococcus*, *Stenotrophomonas*, and fungi_unclassified were crucial to the dynamics of AAs during the solid-state fermentation of traditional Chinese vinegar, which differed from the microbiota that contributed to the formation of AAs. This suggests that different fermentation environments, substrates, and microbiotal structures, and even different geographic locations, affect the type of microbiota that contributes to the formation of AAs.

The genera *Ignatzschineria*, *Weissella*, *Corynebacterium*_1, *Lactococcus*, *Wohlfahrtiimonas*, *Atopostipes*, *Vagococcus*, *Peptostreptococcus*, *Tetragenococcus*, *Bacteroides*, *Kurthia*, unclassified_f__Brucellaceae, *Gulosibacter*, and norank_f__Actinomycetaceae were major producers of the VFs that contributed to the flavor compounds (42 kinds of VFs). In particular, norank_f__Family_XI, *Clostridium_sensu_stricto*_13, *Vagococcus*, *Erysipelothrix*, unclassified_f__Enterobacteriaceae, *Ochrobactrum*, *Alkaliphilus*, and *Tepidanaerobacter* correlated strongly (|*p*| > 0.8) with two alcohols (nos 1–2); *Enterococcus*, *Ignatzschineria*, *Brevibacterium*, *Corynebacterium*_1, *Lactococcus*, *Proteus*, *Helcococcus*, *Paenalcaligenes*, *Saccharopolyspora*, *Wohlfahrtiimonas*, *Gallicola*, norank_f__Bacillaceae, *Atopostipes*, *Vagococcus*, *Enterobacter*, *Peptostreptococcus*, *Tetragenococcus*, *Streptomyces*, *Sphingobacterium*, norank_f__Actinopolysporaceae, norank_p__WS6, *Erysipelothrix*, *Bacteroides*, *Comamonas*, unclassified_f__Enterobacteriaceae, *Leucobacter*, *Kurthia*, *Sporosarcina*, *Curvibacter*, *Nocardiopsis*, norank_o__AKYG1722, *Truepera*, *Empedobacter*, unclassified_f__Brevibacteriaceae, unclassified_c__Bacilli, *Ochrobactrum*, *Globicatella*, *Cronobacter*, unclassified_f__Alcaligenaceae, *Gulosibacter*, *Pisciglobus*, *Alicycliphilus*, *Lentibacillus*, *Moheibacter*, norank_f__Actinomycetaceae, unclassified_f__Rhodobacteraceae, and *Phascolarctobacterium* correlated strongly (|*p*| > 0.8) with 14 esters (nos 3–16); *Weissella*, *Anaerosalibacter*, *Clostridium_sensu_stricto*_15, *Tepidimicrobium*, *Corynebacterium*_1, *Lactococcus*, unclassified_o__Lactobacillales, *Vagococcus*, *Tetragenococcus*, *Prauserella*, *Morganella*, *Cerasibacillus*, and *Amphibacillus* correlated strongly (|*p*| > 0.8) with five pyrazines (nos 17–21); *Providencia*, *Morganella*, norank_p__WS6, *Kurthia*, *Sporosarcina*, *Cerasibacillus*, norank_o__AKYG1722, *Truepera*, and unclassified_f__Rhodobacteraceae correlated strongly (|*p*| > 0.8) with three alkanes (nos 22–24); *Bacillus*, *Enterococcus*, *Corynebacterium*_1, *Lactococcus*, *Proteus*, unclassified_o__Lactobacillales, *Wohlfahrtiimonas*, norank_f__Bacillaceae, *Atopostipes*, *Stenotrophomonas*, *Tetragenococcus*, *Peptoniphilus*, *Bacteroides*, *Leucobacter*, *Ralstonia*, *Nocardiopsis*, *Virgibacillus*, norank_f__Nocardioidaceae, *Ochrobactrum*, *Gulosibacter*, *Pisciglobus*, norank_f__Actinomycetaceae, and *Dietzia* correlated strongly (|*p*| > 0.8) with four aldehydes (nos 25–28); *Bacillus*, *Corynebacterium*_1, *Proteus*, *Wohlfahrtiimonas*, norank_f__Bacillaceae, *Stenotrophomonas*, *Tetragenococcus*, *Peptoniphilus*, *Streptomyces*, *Pseudomonas*, *Kerstersia*, *Acinetobacter*, *Lysinibacillus*, norank_p__WS6, *Erysipelothrix*, *Comamonas*, unclassified_f__Enterobacteriaceae, *Leucobacter*, *Kurthia*, *Sporosarcina*, *Curvibacter*, unclassified_f__Brucellaceae, norank_o__AKYG1722, *Truepera*, *Virgibacillus*, norank_f__Nocardioidaceae, unclassified_f__Brevibacteriaceae, *Ochrobactrum*, *Brevundimonas*, *Elizabethkingia*, *Gulosibacter*, *Rhodococcus*, *Arcobacter*, *Pisciglobus*, norank_f__Actinomycetaceae, unclassified_f__Rhodobacteraceae, *Dietzia*, and *Phascolarctobacterium* correlated strongly (|*p*| > 0.8) with three phenols (nos 29–31); *Weissella*, *Anaerosalibacter*, *Lactobacillus*, *Clostridium_sensu_stricto*_15, *Lactococcus*, unclassified_o__Lactobacillales, *Clostridium_sensu_stricto*_18, *Peptostreptococcus*, *Tetragenococcus*, *Prauserella*, *Morganella*, *Lysinibacillus*, norank_f__Actinopolysporaceae, *Bacteroides*, *Kurthia*, *Sporosarcina*, *Clostridium_sensu_stricto*_11, *Rhodococcus*, *Pisciglobus*, and *Amphibacillus* correlated strongly (|*p*| > 0.8) with six acids (nos 32–37); and *Weissella*, *Bacillus*, *Ignatzschineria*, *Corynebacterium*_1, *Lactococcus*, unclassified_o__Lactobacillales, *Wohlfahrtiimonas*, *Stenotrophomonas*, *Vagococcus*, *Peptostreptococcus*, *Tetragenococcus*, *Peptoniphilus*, *Streptomyces*, *Pseudomonas*, *Erysipelothrix*, *Bacteroides*, *Comamonas*, unclassified_f__Enterobacteriaceae, *Leucobacter*, *Kurthia*, *Curvibacter*, unclassified_f__Brucellaceae, *Virgibacillus*, norank_f__Nocardioidaceae, *Empedobacter*, unclassified_f__Breviba*cteriaceae*, *Ochrobactrum*, *Cronobacter*, unclassified_f__Alcaligenaceae, *Gulosibacter*, *Pisciglobus*, *Alicycliphilus*, norank_f__Actinomycetaceae, *Leuconostoc*, *Dietzia*, and *Phascolarctobacterium* correlated strongly (|*p*| > 0.8) with five other compounds (nos 38–42). The details of the correlations between the microbiota and the flavor components are shown in Fig 4b and 4c. These results are similar to those of Wang et al.[1], who reported that *Acetobacter*, *Lactococcus*, *Lactobacillus*, and *Gluconacetobacer* were important to the dynamics of VFs during the acetic acid fermentation process. In our study, the genera *Ignatzschineria*, *Weissella*, *Corynebacterium*_1, *Lactococcus*, *Wohlfahrtiimonas*, *Atopostipes*, *Vagococcus*, *Peptostreptococcus*, *Tetragenococcus*, *Bacteroides*, *Kurthia*, unclassified_f__Brucellaceae, *Gulosibacter*, and norank_f__Actinomycetaceae contributed the majority of VFs (42 VFs), indicating that they are functional bacterial candidates for the core microbiota in the douchi fermentation process.

### Analysis of the core microbiota in douchi fermentation

**Fig 5:**
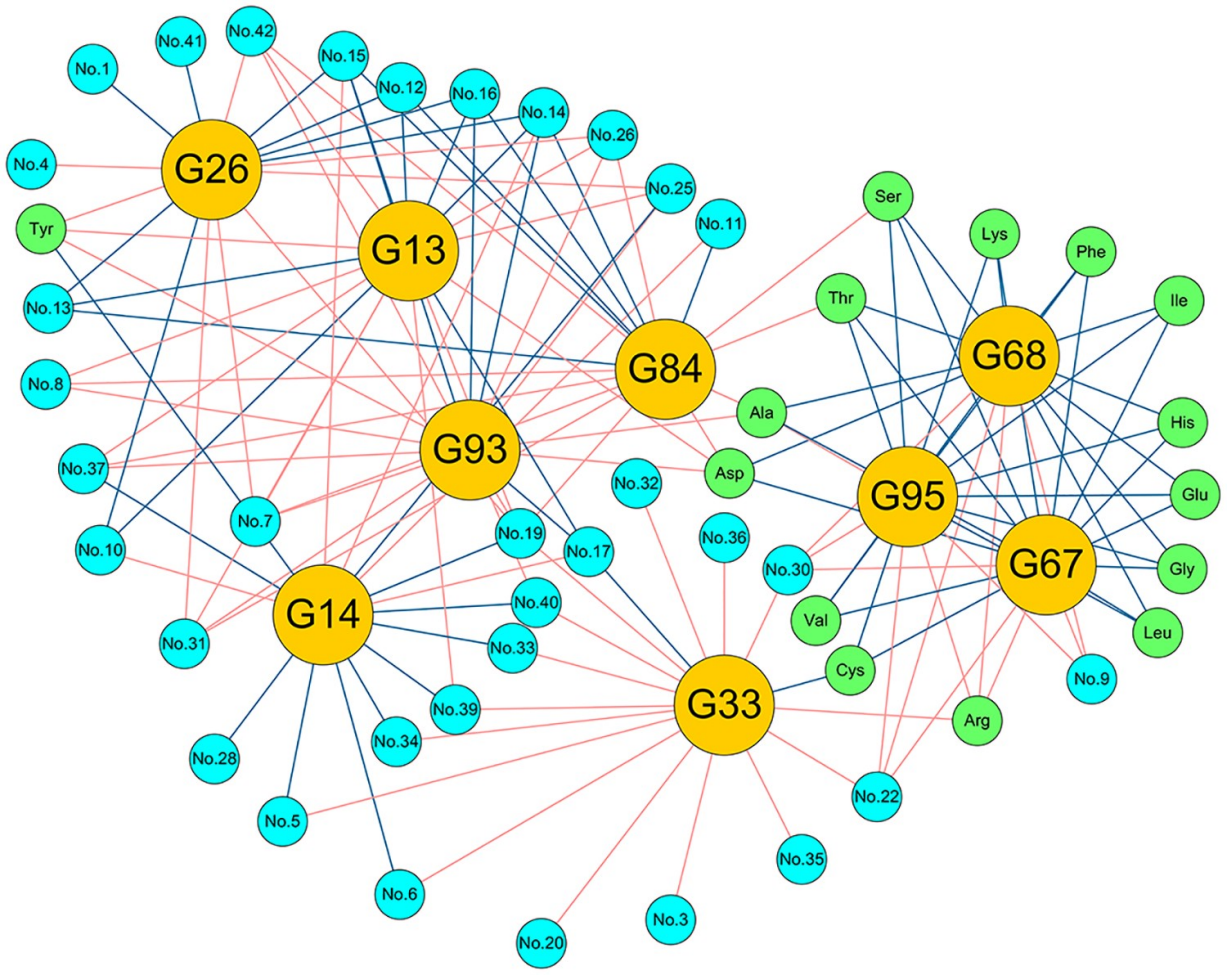
Correlations between core functional microbiota and flavor components in douchi fermentation. The red line between microbiota and flavor components represents positive correlation (p > 0.7) and blue line indicates negative correlation (p < −0.7).

The relationships between the microbiota and the flavor components in douchi fermentation were investigated to identify the functional core microbiota. A total of 49 genera correlated with both AAs and VFs (|*p*| > 0.7). The core microbiota were screened based on the following criteria: (i) production of both AAs and VFs; (ii) VIP_(pred)_ value of at least 1.00; and (iii) correlation with at least 16 flavor components (|*p*| > 0.7). Thus, nine core functional bacteria were identified: *Corynebacterium*_1, *Lactococcus*, *Atopostipes*, *Peptostreptococcus*, norank_o__AKYG1722, *Truepera*, *Gulosibacter*, norank_f__Actinomycetaceae, and unclassified_f__Rhodobacteraceae (Fig 5). These core functional bacteria correlated with both AAs and VFs; specifically, *Corynebacterium*_1, *Lactococcus*, *Atopostipes*, *Peptostreptococcus*, *Gulosibacter*, and norank_f__Actinomycetaceae mainly correlated with VFs, whereas norank_o__AKYG1722, *Truepera*, and unclassified_f__Rhodobacteraceae mainly correlated with AAs. These results are similar to those reported in a previous study[3], which confirms the accuracy of O2PLS modeling and that these six bacteria act as the core functional bacteria in douchi fermentation.

## Conclusions

This study is the first to investigate the key functional flavor-producing bacteria in a traditional Chinese fermented food, douchi. The microbiota and flavor components in the douchi fermentation process were examined with high-throughput sequencing, chromatographic methods, and an O2PLS model, and the key functional bacteria were identified. Our results provide insight into the dynamic changes that occur during the fermentation of douchi. The correlations between the microbial community diversity and the flavor components should be useful in improving the industrial production of douchi and in ensuring the maintenance of the high quality and safety of the product.

## Supporting information

S1 Fig Rarefaction curves for (a) observed species, and (b) Simpson’s diversity indices for samples for bacteria. (DOCX)

S1 Table Evaluation of flavor components and multivariable analysis. (XLSX)

## References

[1] Wang Z-M, Lu Z-M, Shi J-S, Xu Z-H. Exploring flavour-producing core microbiota in multispecies solid-state fermentation of traditional Chinese vinegar. Sci Rep-Uk. 2016;6. 10.1038/srep26818 PMC4886211 27241188

[2] Yang L, Yang H-l, Tu Z-c, Wang X-l. High-Throughput Sequencing of Microbial Community Diversity and Dynamics during Douchi Fermentation. Plos One. 2016;11(12):e0168166 10.1371/journal.pone.0168166 27992473 PMC5167551

[3] Chen C, Xiang JY, Hu W, Xie YB, Wang TJ, Cui JW, et al Identification of key micro-organisms involved in Douchi fermentation by statistical analysis and their use in an experimental fermentation. Journal of Applied Microbiology. 2015;119(5):1324–34. 10.1111/jam.12917 26251195

[4] Park S-E, Yoo S-A, Seo S-H, Lee K-I, Na C-S, Son H-S. GC-MS based metabolomics approach of Kimchi for the understanding of Lactobacillus plantarum fermentation characteristics. Lwt-Food Sci Technol. 2016;68:313–21. 10.1016/j.lwt.2015.12.046

[5] Liu SP, Mao J, Liu YY, Meng XY, Ji ZW, Zhou ZL, et al Bacterial succession and the dynamics of volatile compounds during the fermentation of Chinese rice wine from Shaoxing region. World Journal of Microbiology & Biotechnology. 2015;31(12):1907–21. 10.1007/s11274-015-1931-1 26492888

[6] Piao H, Hawley E, Kopf S, DeScenzo R, Sealock S, Henick-Kling T, et al Insights into the bacterial community and its temporal succession during the fermentation of wine grapes. Front Microbiol. 2015;6. 10.3389/fmicb.2015.00809 PMC4539513 26347718

[7] Trygg J, Wold S. Orthogonal projections to latent structures (O-PLS). Journal of chemometrics. 2002;16(3):119–28.

[8] Wold S, Trygg J, Berglund A, Antti H. Some recent developments in PLS modeling. Chemometrics and intelligent laboratory systems. 2001;58(2):131–50.

[9] Bylesjö M, Eriksson D, Kusano M, Moritz T, Trygg J. Data integration in plant biology: the O2PLS method for combined modeling of transcript and metabolite data. The Plant Journal. 2007;52(6):1181–91. 10.1111/j.1365-313X.2007.03293.x 17931352

[10] Lambert JE, Parnell JA, Han J, Sturzenegger T, Paul HA, Vogel HJ, et al Evaluation of yellow pea fibre supplementation on weight loss and the gut microbiota: a randomized controlled trial. BMC gastroenterology. 2014;14(1):69. 24712378 10.1186/1471-230X-14-69 PMC4234399

[11] Li Y, Liao Q, Lin M, Zhong D, Wei L, Han B, et al An integrated metabonomics and microbiology analysis of host-microbiota metabolic interactions in rats with Coptis chinensis-induced diarrhea. Rsc Adv. 2015;5(97):79329–41.

[12] Dineen S, Aranda Rt, Anders D, Robertson J. An evaluation of commercial DNA extraction kits for the isolation of bacterial spore DNA from soil. Journal of applied microbiology. 2010;109(6):1886–96. 10.1111/j.1365-2672.2010.04816.x 20666869

[13] Liu B, Yuan J, Yiu S-M, Li Z, Xie Y, Chen Y, et al COPE: an accurate k-mer-based pair-end reads connection tool to facilitate genome assembly. Bioinformatics. 2012;28(22):2870–4. 10.1093/bioinformatics/bts563 23044551

[14] You J, Wu G, Ren F, Chang Q, Yu B, Xue Y, et al Microbial community dynamics in Baolige oilfield during MEOR treatment, revealed by Illumina MiSeq sequencing. Appl Microbiol Biot. 2016;100(3):1469–78. 10.1007/s00253-015-7073-4 26496917

[15] Li P, Liang H, Lin W-T, Feng F, Luo L. Microbiota Dynamics Associated with Environmental Conditions and Potential Roles of Cellulolytic Communities in Traditional Chinese Cereal Starter Solid-State Fermentation. Appl Environ Microb. 2015;81(15):5144–56. 10.1128/aem.01325-15 26002897 PMC4495194

[16] Huang X, Liu L, Wen T, Zhu R, Zhang J, Cai Z. Illumina MiSeq investigations on the changes of microbial community in the Fusarium oxysporum f. sp. cubense infected soil during and after reductive soil disinfestation. Microbiological research. 2015;181:33–42. 10.1016/j.micres.2015.08.004 26640050

[17] Tang J, Iliev ID, Brown J, Underhill DM, Funari VA. Mycobiome: Approaches to analysis of intestinal fungi. J Immunol Methods. 2015;421:112–21. 10.1016/j.jim.2015.04.004 25891793 PMC4451377

[18] Igarashi H, Maeda S, Ohno K, Horigome A, Odamaki T, Tsujimoto H. Effect of oral administration of metronidazole or prednisolone on fecal microbiota in dogs. Plos One. 2014;9(9):e107909 10.1371/journal.pone.0107909 25229475 PMC4168260

[19] Marsh AJ, O’Sullivan O, Hill C, Ross RP, Cotter PD. Sequencing-based analysis of the bacterial and fungal composition of kefir grains and milks from multiple sources. Plos One. 2013;8(7):e69371 10.1371/journal.pone.0069371 23894461 PMC3716650

[20] Schloss PD, Westcott SL, Ryabin T, Hall JR, Hartmann M, Hollister EB, et al Introducing mothur: open-source, platform-independent, community-supported software for describing and comparing microbial communities. Appl Environ Microb. 2009;75(23):7537–41. 10.1128/AEM.01541-09 PMC2786419 19801464

[21] Cooper C, Packer N, Williams K. Amino acid analysis protocols: Springer Science & Business Media; 2001.

[22] Caporaso JG, Kuczynski J, Stombaugh J, Bittinger K, Bushman FD, Costello EK, et al QIIME allows analysis of high-throughput community sequencing data. Nature methods. 2010;7(5):335 10.1038/nmeth.f.303 20383131 PMC3156573

[23] Schloss PD, Westcott SL, Ryabin T, Hall JR, Hartmann M, Hollister EB, et al Introducing mothur: open-source, platform-independent, community-supported software for describing and comparing microbial communities. Appl Environ Microbiol. 2009;75(23):7537–41. 10.1128/AEM.01541-09 19801464 PMC2786419

[24] Team RC. R: A language and environment for statistical computing. 2013.

[25] Park S-E, Yoo S-A, Seo S-H, Lee K-I, Na C-S, Son H-S. GC–MS based metabolomics approach of Kimchi for the understanding of Lactobacillus plantarum fermentation characteristics. LWT-Food Science and Technology. 2016;68:313–21.

[26] STANOJLOVIC OP, ZIVANOVIC DP, SUSIC VT. The effect of delta sleep-inducing peptide on the EEG and power spectra in rat. Indian J Physiol Pharmacal. 2000;44(4). 11214497

[27] Loizou C, Pattichis C, Seimenis I, Eracleous E, Schizas C, Pantziaris M, editors. Quantitative analysis of brain white matter lesions in multiple sclerosis subjects: Preliminary findings. 2008 International Conference on Information Technology and Applications in Biomedicine; 2008: IEEE.

[28] Rencher AC. A review of “Methods of Multivariate Analysis”. Taylor & Francis; 2005.

[29] Kim J-H, Kim D-H, Ahn H-J, Park H-J, Byun M-W. Reduction of the biogenic amine contents in low salt-fermented soybean paste by gamma irradiation. Food Control. 2005;16(1):43–9.

[30] Chen TT, Wang MJ, Jiang SY, Xiong SQ, Zhu DC, Wei H. Investigation of the microbial changes during koji-making process of Douchi by culture-dependent techniques and PCR-DGGE. International Journal of Food Science and Technology. 2011;46(9):1878–83. 10.1111/j.1365-2621.2011.02696.x

[31] Panda MK, Sahu MK, Tayung K. Isolation and characterization of a thermophilic Bacillus sp. with protease activity isolated from hot spring of Tarabalo, Odisha, India. Iranian journal of microbiology. 2013;5(2):159 23825735 PMC3696853

[32] Heinen W, Lauwers A, Mulders J. Bacillus flavothermus, a newly isolated facultative thermophile. Antonie van Leeuwenhoek. 1982;48(3):265–72. 10.1007/bf00400386 7125637

[33] Seck EH. Etude de la diversité des procaryotes halophiles du tube digestif par approche de culture: Aix-Marseille; 2017.

[34] Guerzoni ME, Lanciotti R, Cocconcelli PS. Alteration in cellular fatty acid composition as a response to salt, acid, oxidative and thermal stresses in Lactobacillus helveticus. Microbiology. 2001;147(8):2255–64. 11496002 10.1099/00221287-147-8-2255

[35] Niamsup P, Sujaya IN, Tanaka M, Sone T, Hanada S, Kamagata Y, et al Lactobacillus thermotolerans sp. nov., a novel thermotolerant species isolated from chicken faeces. International journal of systematic and evolutionary microbiology. 2003;53:263–8. 10.1099/ijs.0.02347-0 12656183

[36] Garruti DS, Franco MRB, da Silva MAA, Janzantti NS, Alves GL. Assessment of aroma impact compounds in a cashew apple-based alcoholic beverage by GC-MS and GC-olfactometry. Lwt-Food Sci Technol. 2006;39(4):373–8.

[37] McFeeters R. Fermentation microorganisms and flavor changes in fermented foods. Journal of Food Science. 2004;69(1).

[38] Ott A, Fay LB, Chaintreau A. Determination and origin of the aroma impact compounds of yogurt flavor. Journal of agricultural and food chemistry. 1997;45(3):850–8.

[39] Gao X-L, Cui C, Zhao H-F, Zhao M-M, Yang L, Ren J-Y. Changes in volatile aroma compounds of traditional Chinese-type soy sauce during moromi fermentation and heat treatment. Food Science and Biotechnology. 2010;19(4):889–98.

[40] Giri A, Osako K, Okamoto A, Ohshima T. Olfactometric characterization of aroma active compounds in fermented fish paste in comparison with fish sauce, fermented soy paste and sauce products. Food Research International. 2010;43(4):1027–40.

[41] Chen QC, Xu YX, Wu P, Xu XY, Pan SY. Aroma impact compounds in Liuyang douchi, a Chinese traditional fermented soya bean product. International journal of food science & technology. 2011;46(9):1823–9.

[42] Cretenet M, Le Gall G, Wegmann U, Even S, Shearman C, Stentz R, et al Early adaptation to oxygen is key to the industrially important traits of Lactococcus lactis ssp. cremoris during milk fermentation. Bmc Genomics. 2014;15(1):1054 10.1186/1471-2164-15-1054 25467604 PMC4289295

[43] Cho J, Lee D, Yang C, Jeon J, Kim J, Han H. Microbial population dynamics of kimchi, a fermented cabbage product. FEMS microbiology letters. 2006;257(2):262–7. 10.1111/j.1574-6968.2006.00186.x 16553862

[44] Chen T, Wang M, Li S, Wu Q, Wei H. Molecular Identification of Microbial Community in Surface and Undersurface Douchi During Postfermentation. Journal of Food Science. 2014;79(4):M653–M8. 10.1111/1750-3841.12417 24621312

[45] Chen T, Wang M, Jiang S, Xiong S, Zhu D, Wei H. Investigation of the microbial changes during koji-making process of Douchi by culture-dependent techniques and PCR-DGGE. International Journal of Food Science and Technology. 2011;46(9):1878–83. 10.1111/j.1365-2621.2011.02696.x

[46] Johnson JA, El-Dash AA. Role of nonvolatile compounds in bread flavor. Journal of Agricultural and Food Chemistry. 1969;17(4):740–6.

[47] Ye M, Yue T, Yuan Y. Changes in the profile of volatile compounds and amino acids during cider fermentation using dessert variety of apples. Eur Food Res Technol. 2014;239(1):67–77.

[48] Chen C, Xiang J, Hu W, Xie Y, Wang T, Cui J, et al Identification of key micro-organisms involved in Douchi fermentation by statistical analysis and their use in an experimental fermentation. Journal of applied microbiology. 2015;119(5):1324–34. 10.1111/jam.12917 26251195

