# Supplementary material for: Exploring functional core bacteria in fermentation of a traditional Chinese food, Aspergillus-type douchi
Source: PLoS One. 2019 Dec 30;14(12):e0226965. doi: 10.1371/journal.pone.0226965 (PMC6936781; doi:10.1371/journal.pone.0226965)
Supplement: S1 Fig — (DOCX) [file pone.0226965.s001.docx]

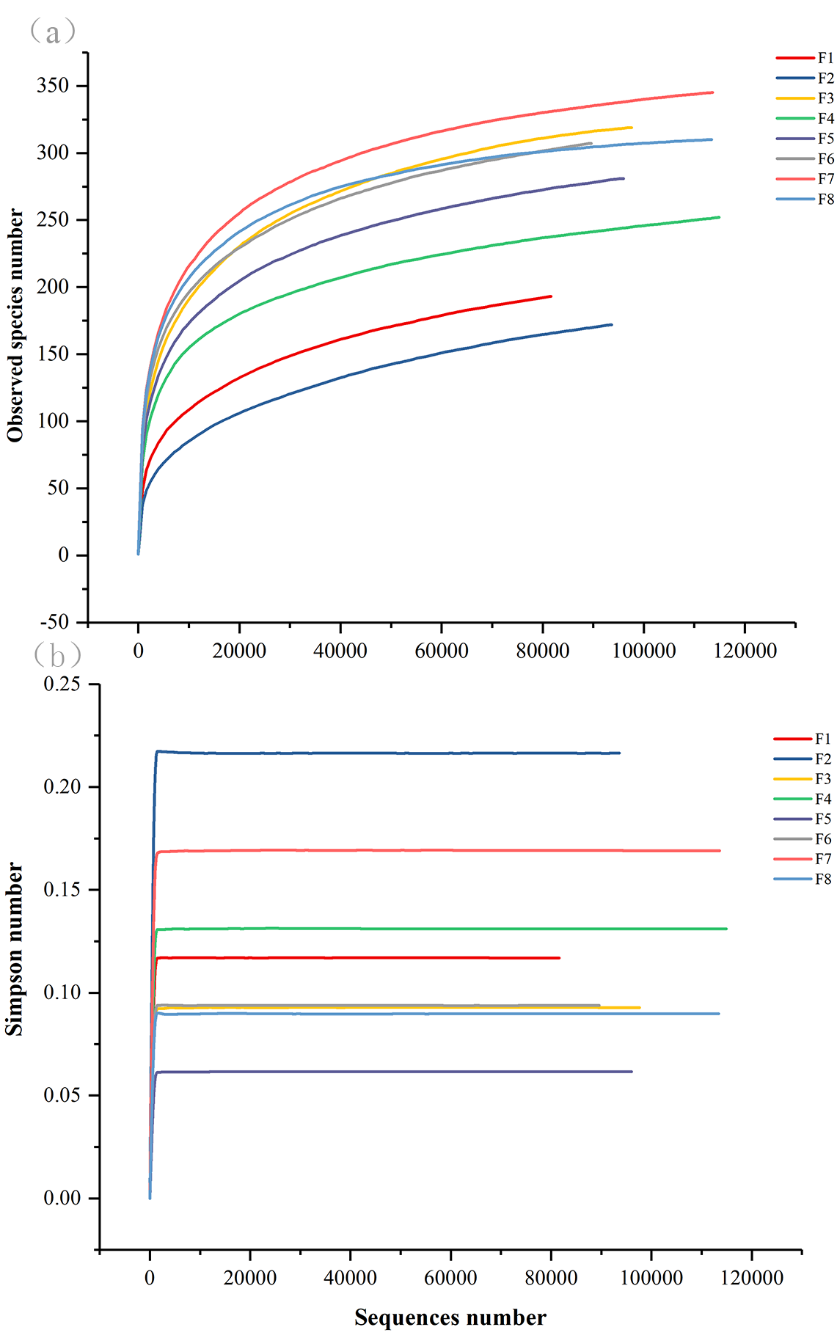


Fig S1 The rarefaction cure for (a) Observed species and (b) Simpson diversity of samples for bacteria
